# HIF-1α-centered metabolic reprogramming in osteoarthritis: cell type-specific effects and intercellular crosstalk among chondrocytes, BMSCs, and macrophages

**DOI:** 10.3389/fimmu.2026.1915008

**Published:** 2026-08-04

**Authors:** Guangxian Liu, Dengju Li, Zhen Wei, Rongzhen Xia, Yichen Liu, Qingchen Zhang, Senbo An, Dawei Wang

**Affiliations:** 1 Department of Orthopaedics, Shandong Provincial Hospital Affiliated to Shandong First Medical University, Jinan, Shandong, China; 2 Department of Joint Surgery, Shandong Provincial Hospital Affiliated to Shandong First Medical University, Jinan, Shandong, China; 3 Department of Rheumatology and Immunology, Shandong Provincial Hospital Affiliated to Shandong First Medical University, Jinan, Shandong, China; 4 Department of Spine Surgery, Central Hospital Affiliated to Shandong First Medical University, Jinan, Shandong, China; 5 School of Pharmacy, Shandong University of Traditional Chinese Medicine, Jinan, China

**Keywords:** bone marrow mesenchymal stromal cell, chondrocyte, HIF-1α, intercellular crosstalk, macrophage polarization, metabolic reprogramming, mitochondrial dysfunction, osteoarthritis

## Abstract

Osteoarthritis (OA) is a systemic metabolic disorder characterized by cartilage degeneration and pathological changes in the “core functional cell populations” in the joints, i.e., chondrocytes, bone marrow mesenchymal stromal cells (BMSCs), and macrophages. In this review, we summarize the current evidence on HIF-1α-centered metabolic reprogramming in OA, with a focus on its cell type-specific effects in chondrocytes, BMSCs, and macrophages, and discuss how HIF-1α-associated intercellular crosstalk may contribute to cartilage degeneration, synovial inflammation, and impaired endogenous repair. While previous reviews mainly addressed HIF-1α-related hypoxia, glycolysis, mitochondrial dysfunction, inflammation, or therapeutic targeting as separate aspects of OA, this review takes a multicellular perspective to emphasize HIF-1α as a cell type-specific metabolic coordinator that connects chondrocyte degeneration, BMSC dysfunction, macrophage polarization, and metabolite-mediated intercellular communication within the OA microenvironment. These core functional cell populations directly regulate cartilage homeostasis, synovial inflammation, and endogenous repair. It is noted that although osteoblasts and osteoclasts are widely recognized as contributors to OA-related subchondral bone remodeling, they are not classified as core functional cell populations in this review due to their primarily restricted functions to bone metabolism-dominated remodeling within the subchondral bone compartment. Under hypoxic, inflammatory, and oxidative-stress conditions, hypoxia-inducible factor-1α (HIF-1α) regulates glycolysis, mitochondrial function, and inflammatory responses. Therefore, while activation to some extent can aid the response of chondrocytes and BMSCs to hypoxemia and induce tissue repair, over-activation is detrimental, leading to tissue damage and aging of BMSCs, as well as an inflammatory M1-like state in macrophages. Therefore, it is recommended that therapeutic strategies should avoid the general inhibition of HIF-1α but focus on cell-specific regulation and targeted intervention of metabolic nodes.

## 1 Introduction

Osteoarthritis (OA) is a widespread age-related degenerative disorder of joints, a cause of many chronic pain syndromes and reduced mobility and largely involved in overall health (1–3). OA was traditionally considered a result of gradual “cartilage damage,” and the focus of pathological research was mainly on changes in the extracellular matrix and a loss of the integrity of articular cartilage (4–6). Although cartilage degeneration remains a central pathological hallmark of OA, a cartilage-centered perspective alone does not fully capture the multicellular and multi-tissue interactions that drive disease initiation and progression (5, 7). OA is now known as a whole-joint disease with numerous abnormal changes in the tissues of the joints, including cartilage, subchondral bone, synovium, bone marrow, blood vessels, and immune cells (8–10). Three functionally important cell populations in this microenvironment are chondrocytes, bone marrow mesenchymal stromal cells (BMSCs), and macrophages; they maintain the homeostatic status of the cartilage matrix, promote endogenously generated repair and lineage differentiation by BMSCs, and modulate the inflammatory environment and immune-metabolic responses via macrophages (11–13).

It should be noted that this classification does not exclude the involvement of osteoblasts and osteoclasts in OA pathogenesis (14). Osteoblasts and osteoclasts are particularly relevant to subchondral bone remodeling, which represents an important pathological component of OA (15). Osteoclasts are derived from the monocyte/macrophage lineage, whereas osteoblasts arise from mesenchymal stem/stromal cells through osteogenic differentiation (16). However, their major OA-related functions are mainly localized to the subchondral bone compartment and are predominantly associated with bone metabolism and remodeling (17). By contrast, the core pathological features in the OA microenvironment are articular cartilage degeneration, synovial inflammation, and endogenous repair (18–20). Therefore, in this review, “core functional cell populations” refer to only chondrocytes, BMSCs, and macrophages, which directly regulate cartilage matrix homeostasis, synovial inflammatory remodeling, and regenerative responses, excluding the bone-metabolism-dominant osteoblasts and osteoclasts (16, 18, 21).

The most featured changes of OA microenvironment include hypoxia, low-grade inflammation, oxidative stress, mitochondrial dysfunction, and metabolic dysregulation. Articular cartilage is an avascular and relatively hypoxic structure, and chondrocytes are adapted to live in a low-oxygen environment (22–24). This concept is supported by classic studies in cartilage biology, showing that the developing growth plate is physiologically hypoxic and that HIF-1α is essential for chondrocyte growth arrest and survival in this avascular tissue (25). Moreover, HIF-1α is required for maintaining glycolytic energy production and extracellular matrix synthesis in epiphyseal chondrocytes under hypoxic conditions (26). With the progress of OA, however, cartilage degeneration, synovial inflammation, subchondral bone sclerosis, abnormal angiogenesis, and reduced nutrient diffusion can be observed in the articular cartilage area. VEGF-A may be an important link between hypoxic signaling and these vascular changes. In healthy adult cartilage, the avascular state is essential for maintaining cartilage homeostasis. During OA progression, however, persistent hypoxic and inflammatory stimulation may enhance HIF-1α-dependent VEGF-A expression, which in turn promotes abnormal angiogenesis, vascular invasion, and neurovascular remodeling within the joint microenvironment (27–29). These modifications create a dynamic hypoxic or pseudo-hypoxic microenvironment to significantly reduce the metabolism and function of the cells (30, 31). Therefore, hypoxia is not only caused by damage to the tissue through a passive route but also acts as a type of regulatory signal that can change the cell’s differentiation status and promote inflammation or regulate cell communication (32, 33).

Hypoxia-inducible factor-1α (HIF-1α) is a master regulator of gene expression in response to hypoxia (34). Under normoxic conditions, Prolyl Hydroxylase Domain proteins (PHDs) hydroxylate HIF-1α, which is then bound by von Hippel-Lindau tumor suppressor protein (pVHL) and degraded by the ubiquitin-proteasome system (34). Under hypoxia, PHD activity is suppressed; as a result, HIF-1α accumulates in the cytoplasm and then moves to the nucleus to form a complex with HIF-1β and promote the expression of genes involved in glycolysis, angiogenesis, cell survival, mitochondrial regulation, redox homeostasis, and inflammation (35). In OA, an increase in the expression of HIF-1α has been observed in cartilage, subchondral bone, synovial macrophages, and BMSCs, and it is well known that HIF-1α is involved in multiple ways in the OA microenvironment (36–38).

The function of HIF-1α in OA is complex, context-dependent, and highly cell-type specific (24). Moderate HIF-1α activation in chondrocytes can help the cells survive under hypoxia, adapt to hypoxia, and be resistant to cellular stress (39). However, prolonged or abnormal HIF-1α activity may increase glycolysis, accumulate lactate, and promote expression of matrix-degrading enzymes, ultimately leading to phenotypic instability (32). Moderate activation of HIF-1α in BMSCs promotes their survival, migration, and differentiation into chondrocytes, all of which are essential for cartilage repair (40). Sustained activation of HIF-1α can impair metabolic flexibility, induce senescence, and increase lactate release, ultimately leading to modified microenvironment in adjacent tissues (32). HIF-1α in macrophages promotes glycolysis-dependent M1-like polarization and the production of inflammatory cytokines (24). Therefore, HIF-1α should not be simply considered either protective or harmful; the biological consequences vary among different cell types, diseases, stages of disease, status of activation, and metabolic environments (41).

Metabolic reprogramming can be employed to study this duality mechanistically. In OA, chondrocytes switch from oxidative phosphorylation to glycolysis and enhance catabolism (42–46). BMSCs are glycolytically dysregulated and senescent, showing impaired chondrogenesis and exhibiting abnormal lineage differentiation (47–49). Macrophages take up a glycolysis-dependent M1-like inflammatory phenotype (50, 51). These cell-specific alterations do not occur independently but are regulated synchronously by metabolites, cytokines, and extracellular vesicles. Mediators of intercellular communication include lactate, succinate, IL-1β, TNF-α, reactive oxygen species (ROS), nitric oxide, and exosomal non-coding RNAs (46, 52). These mediators regulate the effect of HIF-1α-driven metabolic reprogramming in one cell type on the function of adjacent cells, thus forming a self-amplifying pathological network (51, 53, 54).

This review proposes an integrative mechanistic framework for OA progression centered on HIF-1α (24, 38, 55). In this model, HIF-1α is activated in the OA microenvironment by the combination of convergent hypoxic, inflammatory, oxidative, and metabolic stimuli (32, 39, 51, 56). Activated HIF-1α promotes glycolysis, mitochondrial function, mitophagy and redox homeostasis, and metabolite production (57). These processes promote chondrocyte degeneration, regulate BMSC survival and chondrogenesis, promote BMSC senescence and lactate secretion, and drive macrophage M1-like inflammatory polarization (58). Chondrocytes, BMSCs, and macrophages communicate through lactate, succinate, cytokines, and exosomes, forming a multicellular metabolic network that further promotes the development of OA (31, 59). Based on this model, therapeutic strategies should avoid non-selective global inhibition of HIF-1α but focus on cell type-specific modification and targeting of downstream metabolic nodes (38, 55).

Several recent reviews have discussed HIF-1α in OA from the perspectives of hypoxic signaling, glycolytic metabolism, mitochondrial dysfunction, inflammation, autophagy, and therapeutic targeting (24, 32, 55). However, these topics have often been presented as separate mechanisms, and discussion of these mechanisms remain largely chondrocyte-centered. In the present review, we take a broader cellular perspective and organize HIF-1α biology around three functionally important cell populations in the OA joint: chondrocytes, BMSCs, and macrophages. From this viewpoint, HIF-1α is not simply protective or harmful but with varied effects depending on cell type, activation intensity, disease stage, and duration of stimulation.

The main conceptual focus of this review is to connect HIF-1α-driven glycolysis, mitochondrial regulation, lactate and succinate accumulation, cytokine signaling, and exosome-mediated communication within a multicellular metabolic crosstalk model. In this model, HIF-1α activation in one cell population can influence neighboring cells through metabolites and inflammatory mediators, thereby sustaining an inflammation–metabolism feedback loop in the OA microenvironment. This perspective also helps explain why non-selective HIF-1α activation or inhibition may be insufficient, and supports therapeutic strategies based on cell type-specific HIF-1α modulation, targeting of downstream metabolic nodes, macrophage-focused metabolic anti-inflammatory intervention, and metabolic preconditioning of BMSCs. The distinctive focus of the present review compared with previous HIF-1α/OA reviews is summarized in **Table 1**.

**Table 1:** Distinctive focus of the present review compared with previous HIF-1α/OA reviews.

Aspect	Previous review	Present review
Main focus	HIF-1α in hypoxia, glycolysis, mitochondrial dysfunction, inflammation, autophagy, or therapeutic targeting	HIF-1α-centered metabolic reprogramming across chondrocytes, BMSCs, and macrophages
Cellular scope	Mainly chondrocyte-centered or pathway-centered	Defining chondrocytes, BMSCs, and macrophages as core functional cell populations directly linked to cartilage degeneration, endogenous repair, and synovial inflammation
Mechanistic organization	Individual pathways discussed separately	Integration of glycolysis, mitochondrial dysfunction, lactate, succinate, cytokines, ROS, and exosomes into a multicellular metabolic crosstalk network
Interpretation of HIF-1α	Protective or harmful depending on isolated context	Cell type-, stage-, intensity-, and duration-dependent metabolic coordinator
Therapeutic implication	General activation or inhibition of HIF-1α	Cell type-specific HIF-1α modulation, downstream metabolic node targeting, macrophage-focused metabolic anti-inflammatory intervention, and BMSC metabolic preconditioning

## 2 HIF-1α activation in the osteoarthritic joint microenvironment

### 2.1 Hypoxia, pseudohypoxia, and HIF-1α stabilization

Multiple stimuli in the OA joint microenvironment can activate HIF-1α (32, 39, 60). With the progression of OA, damage to the cartilage matrix, synovitis, subchondral bone changes, and altered blood vessel distribution have reduced oxygen supply in the joint (30, 32, 61). Although chondrocytes can survive in a relatively low-oxygen environment, hypoxia caused by diseases differs from that in normal conditions; it often presents as inflammation, oxidative stress, nutrient deficiency, and mechanical damage (31, 62). This pathological condition leads to a prolonged rather than transient activation of HIF-1α (38, 62).

Hypoxia reduces the degradation rate of HIF-1α by inhibiting the PHD/pVHL pathway (63). Reduced oxygen tension inhibits PHD-mediated hydroxylation of HIF-1α, fails to recruit pVHL, ultimately leading to accumulation of HIF-1α. Stabilized HIF-1α then moves to the nucleus and activates genes that promote glycolysis, angiogenesis, cell survival, and metabolic adaptation (64, 65). Among these downstream targets, VEGF-A is particularly relevant to cartilage biology. Previous studies showed that low-oxygen tension markedly increased VEGF mRNA and secreted VEGF protein in epiphyseal chondrocytes, whereas deletion of HIF-1α almost completely abolished this hypoxia-induced VEGF response (27). This study also demonstrated that soluble VEGF isoforms, especially VEGF120 and VEGF164 in mice, were the main isoforms expressed by chondrocytes under low-oxygen conditions. The canonical hypoxia-responsive pathway in OA is also affected by metabolic disturbances, as the activity of PHD is sensitive to oxygen availability and metabolites such as α-ketoglutarate, succinate, and fumarate (66–69).

In addition to actual hypoxia, OA may induce pseudo-hypoxic activation of HIF-1α (70–73). Pro-inflammatory cytokines, such as interleukin-1β (IL-1β) and tumor necrosis factor-alpha (TNF-α), can activate the NF-κB/PI3K/Akt/mTOR and other signaling pathways to promote the transcription, translation, and stabilization of HIF-1α (61, 74–76). Increased ROS caused by damaged mitochondria and the accumulation of succinate in glycolytic or inflammatory cells inhibit PHD activity, thus promoting the stabilization of HIF-1α (39, 77–79). Lactate accumulation enhances extracellular acidification and immunometabolic reprogramming (32, 51, 58). Therefore, HIF-1α in OA should be regarded as a general-purpose sensor for oxygen and other forms of metabolic and inflammatory stresses (24, 32, 39).

**Figure 1:**
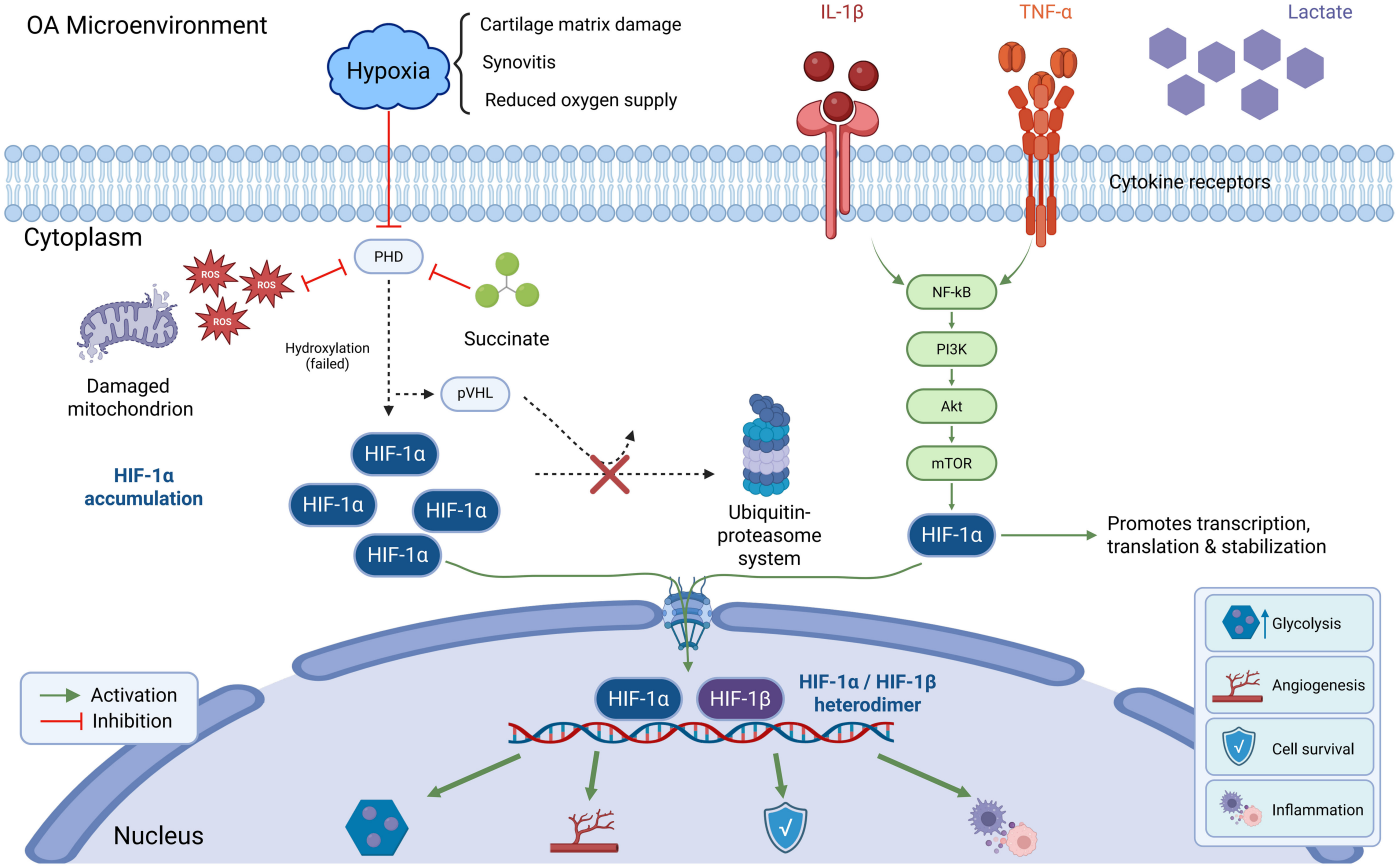
During the pathogenesis of osteoarthritis (OA), progressive cartilage matrix degradation, synovial inflammation, and localized tissue hypoxia collectively establish a sustained hypoxic or pseudo-hypoxic microenvironment and reduce prolyl hydroxylase domain protein (PHD) enzyme activity, thereby inhibiting the hydroxylation of hypoxia-inducible factor-1α (HIF-1α). This impairs recognition by the von Hippel–Lindau tumor suppressor protein (pVHL), disrupts pVHL-mediated ubiquitination, and ultimately prevents proteasomal degradation of HIF-1α—leading to its cytoplasmic accumulation and subsequent nuclear translocation. Concurrently, mitochondrial dysfunction and succinate accumulation elevate reactive oxygen species (ROS) levels, which further inhibit PHD activity and reinforce HIF-1α stabilization. In the meantime, pro-inflammatory cytokines—including interleukin-1β (IL-1β) and tumor necrosis factor-α (TNF-α)—activate the NF-κB/PI3K/Akt/mTOR signaling pathway via cognate cytokine receptors, thereby enhancing HIF-1α transcription, translation, and post-translational stability. Nuclear HIF-1α dimerizes with HIF-1β to form the functional HIF-1 transcription factor complex, which orchestrates the expression of downstream target genes involved in glycolytic metabolism, angiogenesis, cell survival, and inflammatory responses—thereby driving metabolic reprogramming and amplifying chronic inflammation within the OA joint microenvironment.

Upon activation, HIF-1α promotes the expression of glucose transporters and glycolytic enzymes, regulates angiogenic factors such as Vascular Endothelial Growth Factor (VEGF), and interacts with inflammatory pathways and mitochondrial quality control (58, 80–82). These effects are carried out by HIF-1α, which serves as a regulatory hub for hypoxia, inflammation, metabolic disorder, and tissue damage in the osteoarthritic joint (24, 32) (**Figure 1**).

### 2.2 Cell type-specific and dual roles of HIF-1α in OA

HIF-1α plays different biological functions in osteoarthritic-relevant cells, either protective or deleterious, due to its cell type-specific actions (24). The protective role of HIF-1α in chondrocytes was first established in developmental cartilage models. Schipani et al. demonstrated that loss of HIF-1α in growth plate chondrocytes caused cell death in hypoxic cartilage regions, indicating that HIF-1α is indispensable for chondrocyte survival in an avascular microenvironment (25). Pfander et al. further showed that HIF-1α-deficient epiphyseal chondrocytes failed to maintain ATP levels under hypoxia and exhibited reduced aggrecan and type II collagen synthesis, suggesting that HIF-1α supports both metabolic adaptation and extracellular matrix production (26). HIF-1α is required for adaptation to the relatively low-oxygen environment in articular cartilage and chondrocytes (40). Modest HIF-1α activity can support chondrocyte survival, trigger an adaptive stress response, and help maintain cartilage matrix homeostasis (61). In this context, HIF-1α-mediated glycolytic adaptation should be regarded as a physiological requirement rather than a purely pathological event. By promoting anaerobic glycolysis, HIF-1α enables chondrocytes to preserve ATP production in low-oxygen environments and sustain the synthesis of key matrix components, including aggrecan and type II collagen (26). HIF-1α can also promote autophagy and mitophagy to help chondrocytes remove damaged organelles under metabolic stress (39, 83). Similarly, reduced mitochondrial respiration may represent an adaptive mechanism in hypoxic cartilage. Yao et al. reported that suppression of mitochondrial respiration is critical for hypoxia tolerance in fetal growth plate chondrocytes, because excessive mitochondrial oxygen consumption aggravates intracellular hypoxia and promotes chondrocyte death. Therefore, HIF-1α-mediated inhibition of mitochondrial respiration may initially protect chondrocytes by limiting oxygen consumption, although persistent mitochondrial dysfunction and ROS accumulation may become detrimental during OA progression (84).

However, prolonged activation of HIF-1α in OA chondrocytes may become maladaptive under chronic pathological conditions (85). One possible consequence of prolonged HIF-1α activation is sustained VEGF-A production. In developmental cartilage, HIF-1α-dependent VEGF expression contributes to vascular invasion during endochondral ossification. In adult articular cartilage, however, reactivation of this process may be harmful because the maintenance of an avascular cartilage niche is necessary for cartilage homeostasis. Therefore, persistent activation of the HIF-1α/VEGF-A axis may promote pathological angiogenesis and accelerate OA progression (27, 29, 86). These studies indicate that excessive HIF-1α activation can modify cartilage matrix properties, rather than simply enhancing chondrocyte survival. Evidence from articular cartilage further supports the need for precise regulation of the PHD/HIF axis. Cheng et al. reported that PHD2 is highly expressed in the superficial zone of articular cartilage, whereas HIF-1α is mainly detected in the middle-deep zone, whereas conditional deletion of Phd2 in chondrocytes reduces superficial zone thickness, increases chondrocyte hypertrophy, decreases progenitor markers, elevates hypertrophic markers, and increases susceptibility to osteoarthritis-like changes (87). Thus, dysregulated HIF-1α stabilization may contribute to phenotypic instability and pathological differentiation of articular chondrocytes. An overly active glycolysis may result in the accumulation of lactate and extracellular acidification; in the meantime, a redox imbalance also occurs (39, 88). Activation of HIF-1α promotes the expression of matrix-degrading enzymes, such as MMP-13 and ADAMTS-5, and reduces the level of cartilage matrix components, such as type II collagen (89, 90). Therefore, in chondrocytes, the effects of HIF-1α activation vary depending on whether it functions as a short-term adaptive response or as a continuous promoter of metabolic stress and cartilage degradation (41, 91).

HIF-1α also exerts dual effects in BMSCs. Moderate activation of HIF-1α supports cell life, migration, and chondrogenic differentiation (58, 92, 93). It is well known that the BMSCs are under hypoxic and inflammatory conditions in the OA joint and have changed metabolic states (94). Improvement in glycolytic adaptation helps HIF-1α promote survival and matrix synthesis for BMSCs in the initial stage of chondrogenic differentiation (32). Extended HIF-1α activation may reduce metabolic flexibility, interfere with lineage commitment, induce senescence, and promote lactate secretion (95). Therefore, HIF-1α may switch from supporting repair to promoting dysfunctional remodeling (96).

HIF-1α is more regularly associated with inflammatory activation and metabolic reprogramming in macrophages (97, 98). Synovial macrophages under hypoxic and cytokine/lactate/succinate conditions are activated in HIF-1α and switched to a glycolysis-dependent M1-like state (99). HIF-1α promotes the transcription of IL-1β, TNF-α, and iNOS, and thus increases the energy requirements for inflammatory cytokine production (100, 101). These macrophages can increase the severity of synovial inflammation as well as stimulate chondrocyte catabolism and BMSC dysfunction by releasing cytokines, ROS, nitric oxide, and metabolites (50, 102, 103).

The above cell type-specific effects of HIF-1α have different therapeutic applications (38, 41, 55). Global inhibition of HIF-1α may reduce macrophage-driven inflammation, but it may also inhibit chondrocyte adaptation and BMSC-mediated repair (104). Conversely, widespread activation of HIF-1α may support certain repair functions but also exacerbate inflammation (61). Therefore, HIF-1α should be regarded as a context-dependent regulator that requires spatially and temporally precise, cell type-specific modulation.

### 2.3 Context-dependent roles of HIF-2α in cartilage and joint biology

Although this review mainly focuses on HIF-1α-centered metabolic reprogramming, hypoxia-inducible factor-2α (HIF-2α) should also be considered when discussing hypoxia-inducible signaling in cartilage biology. Importantly, HIF-2α does not appear to act as a uniform catabolic factor in all cartilage contexts. Che et al. provided an important mechanistic insight into this issue, showing that HIF-2α can function as a novel inhibitor of chondrocyte maturation in growth plate-related models (105); mechanistically, HIF-2α binds to Runx2, disrupts Runx2/Cbfβ complex formation, promotes Runx2 polyubiquitination and proteasomal degradation, and thereby suppresses Runx2-dependent chondrocyte maturation. This finding is important for the present review because it indicates that HIF-2α should not be simply described as a universal OA-promoting mediator. Rather, its biological effects vary depending on cartilage subtype, developmental stage, inflammatory status, and the difference between physiological maturation and pathological hypertrophy. In developmental cartilage, HIF-2α may restrain Runx2-driven maturation, whereas in adult articular cartilage under inflammatory or degenerative conditions, HIF-2α is more often associated with hypertrophic conversion, matrix degradation, and abnormal osteochondral remodeling (105).

In OA cartilage, recent studies further support a pathological role of HIF-2α in hypertrophic chondrocytes. The IHH–GLI-1–HIF-2α axis has been shown to operate mainly in hypertrophic chondrocytes during OA progression. Under inflammatory conditions, IHH can inhibit HIF-2α degradation through GLI-1 signaling, leading to increased HIF-2α protein expression; in turn, elevated HIF-2α further enhances IHH–GLI-1 signaling, forming a positive feedback loop that regulates downstream hypertrophic factors, matrix-degrading mediators, VEGF expression, and microvascular invasion (106). Therefore, HIF-2α represents an important complementary node to HIF-1α in the OA microenvironment, especially in relation to chondrocyte hypertrophy and osteochondral crosstalk. Accordingly, HIF-2α is discussed here as a context-dependent complementary regulator rather than as part of the central HIF-1α-mediated metabolic framework.

## 3 HIF-1α-mediated metabolic reprogramming in chondrocytes

### 3.1 Glycolytic shift and extracellular matrix degradation

Chondrocytes maintain homeostasis of the extracellular matrix in articular cartilage and are mainly glycolytic under physiological hypoxic conditions (53, 107). However, under OA, hypoxia, IL-1β, TNF-α, ROS, and mechanical stress all promote the stabilization of HIF-1α and enhance the expression of glycolysis-related genes (39, 108). HIF-1α directly or indirectly promotes the expression of glucose transporters and glycolytic enzymes, such as GLUT1/SLC2A1, HK2, PFKFB3, PKM2, and LDHA (109–111). Therefore, chondrocytes shift further toward glycolysis and away from balanced mitochondrial oxidative phosphorylation (47, 88).

The shift toward glycolysis may be a compensatory adaptation that helps chondrocytes survive and produce extracellular matrix in a hypoxic environment at first (53, 108, 109). Several original experimental studies support the view that HIF-1α-dependent glycolysis can preserve chondrocyte viability and matrix-producing activity under hypoxic conditions. Pfander et al. reported that HIF-1α-deficient epiphyseal chondrocytes failed to maintain ATP levels under hypoxia, showing reduced aggrecan and type II collagen expression and demonstrating the requirement of HIF-1α for anaerobic glycolysis and extracellular matrix synthesis (26). In cartilage repair-related models, icariin-induced HIF-1α activation promoted chondrocyte proliferation, expression of Sox9, Col2α1, and aggrecan, extracellular matrix production, and osteochondral defect repair, while HIF-1α deletion weakened these effects (112). Consistently, icariin was later shown to enhance chondrocyte vitality and extracellular matrix synthesis by increasing HIF-1α expression and anaerobic glycolysis-related enzymes, including GLUT1, G6PD, PGK1, and PDK1 (113). These findings suggest that moderate HIF-1α-driven glycolytic adaptation may be beneficial for chondrocyte survival and matrix synthesis, whereas prolonged activation in OA may contribute to catabolic remodeling. However, prolonged activation of glycolysis is detrimental due to an increase in lactate and other causes (114). Activation of HIF-1α in OA cartilage is associated with an increase in MMP-13 expression and a decrease in type II collagen expression, while persistent HIF-1α-driven glycolysis may promote a catabolic chondrocyte phenotype (88). This interpretation is consistent with previous reviews summarizing the dual role of HIF-1α in OA metabolism and cartilage homeostasis (24, 32).

The catabolic phenotype of OA chondrocytes may also be influenced by HIF-2α-dependent signaling. However, in light of the findings by Che et al. (105), HIF-2α should be interpreted in a context-dependent manner rather than as a simple catabolic switch. In adult OA cartilage, HIF-2α appears to be more closely linked to hypertrophic differentiation and matrix-degrading activity, promoting COL-X, MMP13, ADAMTS4, VEGF, and Runx2-related pathological programs, thereby connecting hypoxia-inducible signaling with cartilage matrix loss and osteochondral remodeling (106, 115).

There are also associations between glycolysis and extracellular matrix degradation (61, 116, 117). Glycolytic intermediates can regulate inflammatory signaling, oxidative stress and ferroptosis, NADPH production, and susceptibility to lipid peroxidation and iron metabolism (118–121). Thus, HIF-1α-mediated glycolysis may function as an adaptive metabolic route and simultaneously as a signaling platform that links metabolic stress with matrix degradation and ferroptosis-related chondrocyte injury (122–125).

### 3.2 Mitochondrial dysfunction, oxidative stress, and mitophagy

Although chondrocytes are mainly glycolytic, mitochondria are required for redox regulation, apoptosis control, calcium signaling, and responses to stress (32, 126). OA chondrocytes often show mitochondrial dysfunction, such as a reduced mitochondrial membrane potential, defective ATP synthesis, increased level of ROS, and impaired mitochondrial quality control (77). These processes are regulated by HIF-1α through mitochondrial metabolism, oxidative phosphorylation, and mitophagy (24).

HIF-1α reduces mitochondrial oxygen consumption by altering the cellular mode of energy production under hypoxia to limit excessive ROS generation (127). HIF-1α can also promote mitophagy and help identify damaged mitochondria (128, 129). For instance, stabilization of HIF-1α by the PHD inhibitor Dimethyloxalylglycine (DMOG) promotes mitophagy, reduces chondrocyte apoptosis and senescence, and may thus mitigate cartilage damage in model OA (83, 130). Furthermore, HIF-1α knockdown is also associated with mitochondrial dysfunction and chondrocyte damage (57).

However, when mitochondrial damage exceeds the capacity for mitophagy, dysfunctional mitochondria produce excessive ROS (131), which further stabilizes HIF-1α by inhibiting PHD activity and activating inflammatory pathways to form a self-amplifying HIF-1α/ROS feedback loop (132). This loop may trigger inflammasome-related pathways, such as NLRP3 signaling, increase the secretion of inflammatory cytokines, and cause chondrocyte apoptosis and senescence and matrix degradation (133–135). Therefore, therapeutic strategies that aim to inhibit HIF-1α should be combined with those that restore mitochondrial quality control rather than simply inhibiting HIF-1α activity (136).

### 3.3 Phenotypic transformation, senescence, and ferroptosis

One feature of OA is chondrocyte phenotypic instability (106). Healthy articular chondrocytes are anabolic, producing type II collagen and aggrecan, whereas OA chondrocytes may be catabolic, hypertrophic-like, senescent, apoptotic, or ferroptotic (24, 33). These changes are closely associated with HIF-1α-mediated metabolic reprogramming (38, 61, 137).

Increased glycolysis promotes transcription of hypertrophic and catabolic genes, including *COL10A1* and *MMP-13* (36, 138, 139). HIF-1α-induced metabolic changes may modify transcriptional programs for chondrocyte differentiation and extracellular matrix remodeling (55, 83). However, under certain circumstances, HIF-1α can also maintain cartilage homeostasis by inhibiting catabolic gene expression and restricting NF-κB signaling; thus, chondrocyte-specific deletion of HIF-1α has been shown to accelerate OA progression (38, 140). Numerous studies have demonstrated that the effects of HIF-1α vary with the intensity and duration of activation and inflammation (38, 141).

Chronic metabolic stress can induce more cell senescence and ferroptosis (142). OA chondrocytes that are chronically inflamed and stressed by oxidants show an enhanced expression of senescent-associated genes and reduced synthesis activity (143). HIF-1α can delay senescence by promoting mitophagy, but it may also lead to senescence-associated dysfunction when combined with high ROS production and continuous glycolysis (142). HIF-1α in inflammatory OA may promote ferroptosis by increasing the uptake of iron via transferrin receptor 1 (TFRC), thereby enhancing lipid peroxidation and damaging chondrocytes (24, 144). Single-cell transcriptomics studies have shown that HIF-1α participates in hypoxia-induced chondrocyte lineage plasticity and hypertrophic transformation during OA progression (145, 146).

HIF-1α-mediated metabolic reprogramming of chondrocytes is generally regarded as context-dependent, with moderate activation promoting hypoxic adaptation and mitochondrial quality control, whereas prolonged activation in the presence of inflammation and oxidative stress leads to matrix degradation, phenotypic instability, senescence, ferroptosis, and functional decline (46).

### 3.4 HIF-1α-dependent VEGF-A elevation and pathological angiogenesis

In addition to its role in glycolytic adaptation and mitochondrial regulation, HIF-1α can also regulate VEGF-A expression. This pathway is important in cartilage biology due to the involvement of VEGF-A in vascular invasion during endochondral ossification (27). Under physiological developmental conditions, this process is required for the replacement of cartilage by bone; in adult articular cartilage, however, excessive or persistent activation of this pathway may become pathological, as mature cartilage normally remains avascular and aneural.

Recent evidence suggests that this mechanism may also be involved in OA progression. Sustained HIF-1α accumulation was reported to increase VEGF expression in articular cartilage and synovium, promote pathological angiogenesis, and disturb the avascular hypoxic cartilage niche (29). These changes were accompanied by synovial inflammation, matrix loss, and accelerated structural degeneration, suggesting that persistent HIF-1α/VEGF-A activation may amplify joint damage in a compartment- and stage-dependent manner.

VEGF signaling may also contribute to the symptomatic features of OA. VEGF-A can activate both VEGFR1 and VEGFR2 receptors, which appear to play different roles in OA pathology. VEGFR1 signaling is more closely associated with nociceptive sensitization and pain transmission, whereas VEGFR2 signaling is more closely related to cartilage degeneration and catabolic remodeling. Thus, HIF-1α-dependent VEGF-A elevation may contribute to OA progression not only by promoting abnormal angiogenesis, but also by participating in pain-related neurovascular signaling and cartilage catabolism (28).

## 4 Role of HIF-1α in BMSC fate regulation

### 4.1 Metabolic control of chondrogenesis and aberrant differentiation

BMSCs are considered optimal joint repair cells due to their multiple differentiation pathways into chondrogenic, osteogenic, or adipogenic cells by changing their microenvironment (147–149). OA often impairs the repair function of BMSCs (58). BMSCs in the damaged joint are exposed to hypoxia, inflammatory cytokines, oxidative stress, modified matrix stiffness, and metabolic stress (93, 150). HIF-1α is a master regulator that helps BMSCs adapt to this adverse microenvironment (151).

Early in chondrogenic differentiation, glycolysis is required to some extent for BMSC proliferation, maintenance, and production of cartilage matrix (152). Moderate activation of HIF-1α promotes glycolytic adaptation and supports the growth of BMSCs in hypoxia (32). Therefore, the subsequent modifications are necessary to alleviate the relative hypoxia of the joint cavity and cartilage repair niche (153). With insufficient adaptation to hypoxia, the BMSCs undergo apoptosis or lose their differentiation ability after migration and transplantation into the OA joint (152).

HIF-1α promotes chondrogenic differentiation (107). Modest induction of HIF-1α enhances the expression of chondrogenic factors and creates a favorable metabolic state for the synthesis of cartilage matrix (24, 92, 154). Therefore, HIF-1α may alleviate BMSC-mediated repair (78), enable the survival and migration of BMSCs, and induce chondrogenic differentiation in a relatively unfavorable microenvironment (152), suggesting that HIF-1α should not be considered only a disease factor in BMSCs (155).

However, the duration and level of HIF-1α activation determine the damage level in the cells (156). Sustained hypoxia and inflammation in osteoarthritis may lead to prolonged HIF-1α activation (157). Overexpression of HIF-1α-driven glycolysis reduces metabolic flexibility, i.e., the ability of BMSCs to shift between glycolysis and oxidative phosphorylation during differentiation (32). Chondrogenesis requires an organized metabolic program that is not driven by excessive glycolysis (92, 158), while sustained activation of HIF-1α may prevent the appropriate differentiation of BMSCs (159, 160).

Metabolic disorder may modify the transcriptional and epigenetic program of differentiation (107, 161). Glycolytic intermediates and mitochondrial metabolites can regulate histones, DNA methylation, transcription factor activity (85). Although these mechanisms have not been identified in OA-associated BMSCs, it can be expected that metabolic reprogramming would regulate their differentiation. Therefore, HIF-1α-mediated metabolic regulation may determine whether BMSCs are effectively chondrogenically differentiated, aberrantly differentiated, or functionally impaired (160).

Therefore, the goals are to induce HIF-1α at a low level, promote the survival of BMSCs and their differentiation into chondrocytes, and prevent over-activation at high levels (83, 159). These two mechanisms are particularly applicable to regenerative medicine (92). Under weak HIF-1α activity, the BMSCs cannot adjust and survive in hypoxia (158). HIF-1α activity would also be enhanced under this condition, leading to impaired differentiation state of BMSCs (159). Therefore, the therapeutic aim should control HIF-1α activation that supports BMSC viability and chondrogenesis without causing metabolic exhaustion (92).

### 4.2 BMSC senescence and metabolite-mediated microenvironmental effects

BMSC senescence is a particular cause for the impaired repair function in OA (161, 162). Chronic inflammation, oxidative stress, mitochondrial dysfunction, and nutrient deprivation can induce premature senescence in BMSCs (163). Senescent BMSCs have reduced proliferation and differentiation, abnormal metabolism, and elevated levels of inflammatory factors (158, 161). HIF-1α promotes glycolysis, regulates mitochondrial function, increases ROS level, and participates to some extent in NAD^+^-dependent signaling (164).

Sustained activation of HIF-1α can promote BMSC senescence through glycolysis and reduce the balanced mode of mitochondrial oxidative phosphorylation (165). Metabolic changes may result in a lack of energy, an increase in ROS, and thus a reduction in the efficacy of stress-response mechanisms (166). Senescent BMSCs may release senescence-associated secretory phenotype (SASP) factors, such as IL-6, IL-1β, chemokines, and matrix-degrading enzymes (167). These mediators can further autocrinely impair the function of BMSCs and paracrinely promote chondrocyte death and macrophage activation (98).

HIF-1α-mediated glycolysis in BMSCs could alter the microenvironment by releasing lactate and promote senescence (58). Lactate is a high-yield product of glycolysis (168). When HIF-1α increases the expression of glycolytic enzymes and glucose transporters in BMSCs, glycolytic flux rises, leading to the production of lactate and extracellular release (32, 169). This route should not be regarded merely for waste products (170); lactate is also a signal molecule that can influence nearby chondrocytes and macrophages (171).

BMSC-derived lactate may increase glycolytic metabolism and cause metabolic disorders in chondrocytes (172). Hypoxia and inflammation increase lactate production in the cartilage, leading to decreased extracellular pH, altered redox state, and catabolism (173). Thus, BMSC metabolism can directly affect chondrocyte behavior (114).

BMSC-derived lactate can be beneficial for macrophages in the environment of OA (88, 174). Lactate can stabilize HIF-1α in macrophages and promote glycolysis-dependent M1-like polarization (175). HIF-1α activation in BMSCs can thus promote inflammation indirectly by increasing the supply of lactate to macrophages (32). M1-like macrophages then release IL-1β, TNF-α, ROS, nitric oxide, and matrix-degrading factors to further damage the cartilage and inhibit BMSC chondrogenesis (50). A positive feedback loop has thus been established as follows: HIF-1α stimulates glycolysis and lactate production in BMSCs, and lactate further activates HIF-1α and induces inflammatory polarization of macrophages; subsequently, cytokines secreted by macrophages exacerbate BMSC dysfunction and chondrocyte degeneration (176, 177).

**Figure 2:**
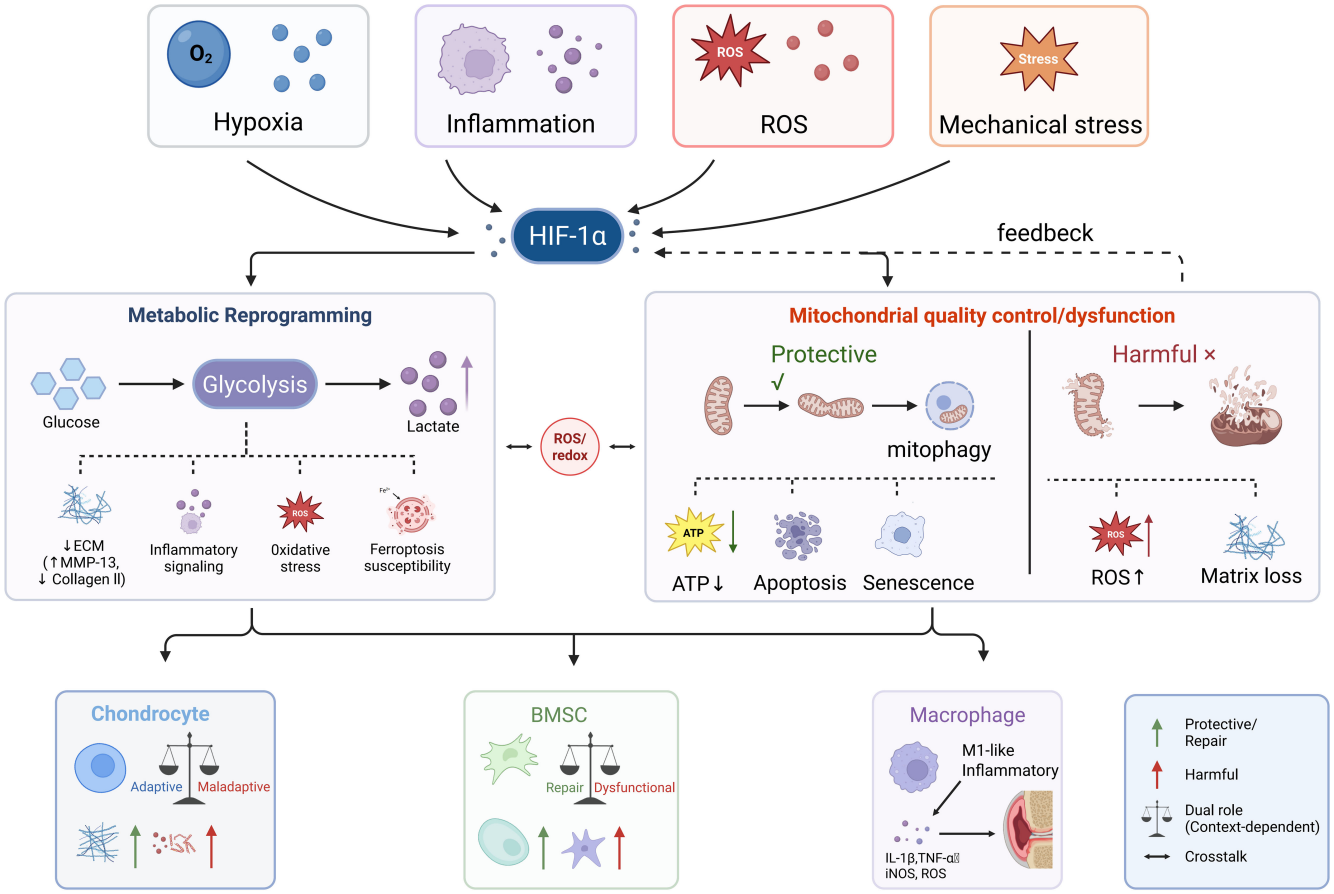
HIF-1α serves as a central regulatory node mediating the dual, context-dependent roles of metabolic reprogramming and mitochondrial homeostasis dysregulation in the osteoarthritic (OA) joint microenvironment. Hypoxia, pro-inflammatory cytokines, reactive oxygen species (ROS), and aberrant mechanical loading collectively promote HIF-1α protein stabilization and transcriptional activation. This leads to upregulated glycolytic flux and intracellular lactate accumulation in chondrocytes and bone marrow mesenchymal stromal cells (BMSCs), thereby modulating inflammatory signaling pathways, extracellular matrix (ECM) catabolism, ferroptosis susceptibility, and cell fate decisions—largely through redox state modulation. Under physiological hypoxic conditions, moderate HIF-1α activation supports chondrocyte adaptation to low oxygen, enhances BMSC viability, facilitates chondrogenic differentiation, and maintains mitochondrial quality control via induction of mitophagy. In contrast, chronic or excessive HIF-1α activation drives pathological lactate efflux, disrupts cellular bioenergetics, amplifies oxidative stress, accelerates cellular senescence, and contributes to progressive ECM degradation. In macrophages, HIF-1α reinforces glycolysis-dependent M1-like polarization, resulting in elevated secretion of IL-1β, TNF-α, inducible nitric oxide synthase (iNOS), and ROS—thereby exacerbating cartilage destruction and impairing BMSC regenerative capacity. Collectively, these findings underscore that HIF-1α exerts cell type–specific, intensity-, and duration-dependent biphasic effects in OA: protective at physiological levels but detrimental under sustained pathological activation.

Therefore, BMSCs should be regarded as both repair cells and active metabolic units in the OA microenvironment (178). Under moderate activation of HIF-1α, BMSCs promote repair by increasing cell viability and chondrogenesis (179); under prolonged activation of HIF-1α, they can act as sources of lactate and inflammatory signals that promote the progression of OA (53). Given the above considerations, BMSC-based therapy requires precise metabolic regulation (179) (**Figure 2**).

## 5 HIF-1α-driven macrophage polarization and inflammatory metabolism

### 5.1 Glycolysis-dependent M1-like polarization

Macrophages are key mediators of synovial inflammation in OA (18). Although OA is not traditionally considered an autoimmune disease, low-grade chronic inflammation has now been identified as one of the causes for pain, cartilage degeneration, and the advancement of the disease (180). Synovial macrophages exist in various states. M1-like macrophages produce pro-inflammatory cytokines and matrix-degrading factors, and M2-like macrophages are associated with the resolution of inflammation and tissue repair (181). These two phenotypes are determined by an abnormal metabolic state (182).

HIF-1α is a hub of metabolism that promotes M1-like polarization (183). Pro-inflammatory M1-type macrophages are generally glycolytic and thus produce ATP quickly to support cytokine release (184). HIF-1α increases the expression of glucose transporters and glycolytic enzymes to promote glycolysis (98). M2-like macrophages are non-inflammatory and rely more on fatty acid oxidation to support tissue repair. Therefore, HIF-1α-mediated glycolysis shifts macrophages toward a pro-inflammatory state (185).

Hypoxia, cytokines, ROS, lactate, and succinate can activate HIF-1α in OA synovium macrophages (39). Activated HIF-1α increases the production of IL-1β, TNF-α, iNOS, and other inflammatory substances (79). These cytokines further enhance chondrocyte catabolism and suppress BMSC chondrogenesis (53). HIF-1α-driven glycolysis in macrophages also produces metabolites that promote inflammation (104), thus not only just helping activate macrophages, but also providing metabolic support for prolonged inflammation (50).

Regulatory factors that inhibit HIF-1α can suppress macrophage glycolysis and M1-like polarization (185). SET Domain Containing 2 (SETD2) suppresses HIF-1α and thus reduces glycolysis and inflammatory macrophage polarization (186), indicating that HIF-1α may be a target for drug development in the inflammation of macrophages (83). However, as HIF-1α plays protective roles in chondrocytes and BMSCs, macrophage-specific targeting may be more suitable than whole-body inhibition (61).

HIF-1α-mediated macrophage activation impacts other cell types via cytokines, ROS, nitric oxide, and metabolites (32). IL-1β and TNF-α released by M1-like macrophages can stabilize HIF-1α in chondrocytes and BMSCs, thereby inducing hypoxia-like metabolic reprogramming under non-hypoxic conditions (187). ROS and nitric oxide generated by macrophages can damage mitochondria in chondrocytes and BMSCs, further promoting glycolysis and HIF-1α activation (24). Therefore, macrophages act as inflammatory amplifiers that drive widespread degenerative damage in the joint through local metabolic stress (188).

### 5.2 Macrophage-derived metabolites and inflammatory amplification

Macrophage-derived metabolites are key mediators of inflammatory amplification in OA (189). Among these metabolites, succinate and lactate are particularly relevant because they directly connect metabolic reprogramming to HIF-1α stabilization and inflammatory signaling (190).

Tricarboxylic acid cycle reorganization in glycolytic M1-like macrophages enhances succinate accumulation (191). Succinate inhibits PHD activity to promote HIF-1α stabilization and thus forms a self-enhancing positive feedback loop (192). Stabilized HIF-1α also promotes glycolysis and IL-1β production, thus strengthening the pro-inflammatory phenotype (193). Succinate can act on adjacent cells, such as chondrocytes and BMSCs, to enhance the activity of HIF-1α and glycolytic reprogramming (192). Therefore, succinate acts as an end-product for internal metabolic regulation and simultaneously triggers inflammation from the outside (46).

Lactate is another key immunometabolic intermediate (194). Although lactate has different effects in different tissues depending on the context, it may help sustain macrophage glycolysis and M1-like polarization in the inflammatory OA microenvironment (195). Lactate from BMSCs, chondrocytes, or macrophages can stabilize HIF-1α and promote a pro-inflammatory metabolic state (196). Therefore, multiple OA-associated cell types have increased glycolysis and lactate production (114), and a lactate-rich microenvironment arises to promote immunometabolic dysfunction (195).

Macrophage-derived cytokines work with metabolites to enhance OA-associated damage (32). IL-1β and TNF-α activate HIF-1α in chondrocytes and BMSCs, induce glycolytic reprogramming, inhibit matrix synthesis, and promote the expression of catabolic genes (99). These cytokines also increase ROS generation and damage mitochondria to further promote the activation of HIF-1α (197). Therefore, macrophage-derived metabolites and cytokines form an integrated inflammation-metabolism circuit (32).

This proposed circuit may have several pathological consequences. First, it induces the production of catabolic enzymes by chondrocytes that degrade cartilage matrix. Second, it inhibits BMSC chondrogenic differentiation and repair function. Third, it maintains synovial inflammation by promoting M1-like polarization of macrophages (32). Fourth, it may contribute to a potentially self-reinforcing feedback loop involving hypoxia, HIF-1α signaling, glycolysis, lactate, succinate, and inflammatory cytokines (198). Therefore, these interconnected processes suggest that targeting a single inflammatory mediator may be insufficient and that combined intervention at multiple inflammatory and metabolic nodes may be required.

## 6 Intercellular metabolic crosstalk among chondrocytes, BMSCs, and macrophages

### 6.1 Lactate, succinate, and cytokine feedback loop

The OA microenvironment should be regarded as a multicellular metabolic network, rather than a group of isolated cell defects (32). Chondrocytes, BMSCs, and macrophages communicate via metabolites and cytokines, and at the center of this communication network is HIF-1α (38).

Hypoxia and inflammation lead to HIF-1α-mediated metabolic shift toward glycolysis and lactate production in chondrocytes and BMSCs (199). Lactate can be taken up by macrophages or act through signaling pathways to stabilize HIF-1α and promote glycolysis-dependent M1-like polarization (200), and in turn, M1-like macrophages release IL-1β, TNF-α, ROS, nitric oxide, and succinate (201). These factors inhibit chondrocytes and BMSCs, thus activating HIF-1α and causing mitochondrial dysfunction, metabolic reprogramming, and catabolism (187).

Succinate is the substrate of a metabolic pathway. Succinate inhibits PHD activity and therefore promotes HIF-1α stabilization in macrophages (202). Extracellular succinate can also affect nearby cells and enhance inflammatory signaling (203). Succinate-induced stabilization of HIF-1α in chondrocytes and BMSCs promotes glycolysis and impairs mitochondrial function (204). Thus, succinate serves as a metabolic amplifier for the HIF-1α pathway.

Cytokines also extend this HIF-1α-centered inflammation–metabolism feedback loop. IL-1β and TNF-α generated by macrophages or inflamed synovial cells can stabilize HIF-1α in chondrocytes and BMSCs to induce pseudo-hypoxic glycolytic reprogramming (99), reduce cartilage matrix synthesis, induce expression of catabolic enzymes, and inhibit BMSC chondrogenesis. HIF-1α-induced glycolysis increases the generation of inflammatory factors and therefore establishes an inflammation-metabolism feedback loop (99).

The above network may explain why OA progression is difficult to stop. The HIF-1α/VEGF-A axis may further broaden this network by involving vascular and neural components. Studies show that increased VEGF-A can promote abnormal angiogenesis and vascular invasion, while VEGF/VEGFR signaling may influence synovial inflammation, sensory nerve activation, and pain transmission (28, 29). Therefore, HIF-1α-driven VEGF-A elevation provides a possible connection between hypoxic-metabolic stress, structural degeneration, and symptomatic OA. Chondrocyte degeneration releases stress signals that activate macrophages (32). Activated macrophages produce cytokines and metabolites that promote chondrocyte catabolism and BMSC dysfunction (50), and in turn, dysfunctional BMSCs release lactate and inflammatory factors that promote the polarization of macrophages (41). All the cell types either respond to or promote the metabolism of other cell types. Therefore, HIF-1α-centered metabolic crosstalk provides an overarching mechanism for OA progression (205).

### 6.2 Extracellular vesicle-mediated transfer of metabolic and regulatory signals

Extracellular vesicles, particularly exosomes, may contribute to intercellular communication within the OA joint microenvironment by transferring proteins, lipids, microRNAs (miRNAs), long non-coding RNAs, messenger RNAs, and other metabolism-related regulatory molecules to recipient cells (206–208). Chondrocytes, bone marrow mesenchymal stromal cells (BMSCs), macrophages, synovial fibroblasts, and other joint-resident cells can release extracellular vesicles. The biological effects of these vesicles are likely to depend on the donor cell type, cargo composition, recipient cell state, and local pathological context (206, 208).

Among the currently available studies, inflammatory fibroblast-like synoviocyte-derived exosomes provide relatively direct evidence linking extracellular vesicle-mediated communication to HIF-1α-associated metabolic reprogramming in OA. Exosomes derived from inflammatory fibroblast-like synoviocytes were reported to enhance glycolysis and M1-like polarization in macrophages. Pharmacological inhibition of glycolysis or genetic and pharmacological inhibition of HIF-1α attenuated the macrophage inflammatory response induced by these exosomes, while intra-articular administration of inflammatory exosomes exacerbated synovitis and experimental OA (200). These findings support a local fibroblast-like synoviocyte–macrophage immunometabolic communication pathway within the OA joint.

Importantly, extracellular vesicle-mediated communication in OA is not uniformly pathogenic. Exosomes derived from hypoxia-preconditioned adipose-derived stromal cells have been reported to attenuate chondrocyte inflammaging and reduce OA progression in preclinical models (209). Dental pulp stromal cell-derived exosomal miR-31 may promote chondrocyte autophagy through inhibition of mTOR signaling and exert chondroprotective effects in experimental OA (210). Exosomes derived from bone marrow and adipose-derived mesenchymal stromal cells have also shown source-dependent differences in their cartilage-protective effects in an OA mouse model (211). These observations indicate that the biological consequences of extracellular vesicle-mediated communication depend on the donor cell phenotype, molecular cargo, recipient cell type, and disease context rather than representing a uniformly pathological process.

Current evidence mainly supports extracellular vesicle-mediated communication among spatially separated cells within the local or intra-articular OA microenvironment. However, most mechanistic evidence has been obtained from *in vitro* experiments and preclinical animal models, and direct evidence that extracellular vesicles mediate long-range or systemic propagation of HIF-1α-centered metabolic dysfunction in human OA remains limited (206–208). Therefore, extracellular vesicle-mediated signaling should currently be regarded as a plausible component of local metabolic and inflammatory crosstalk rather than an established mechanism of systemic disease propagation.

**Figure 3:**
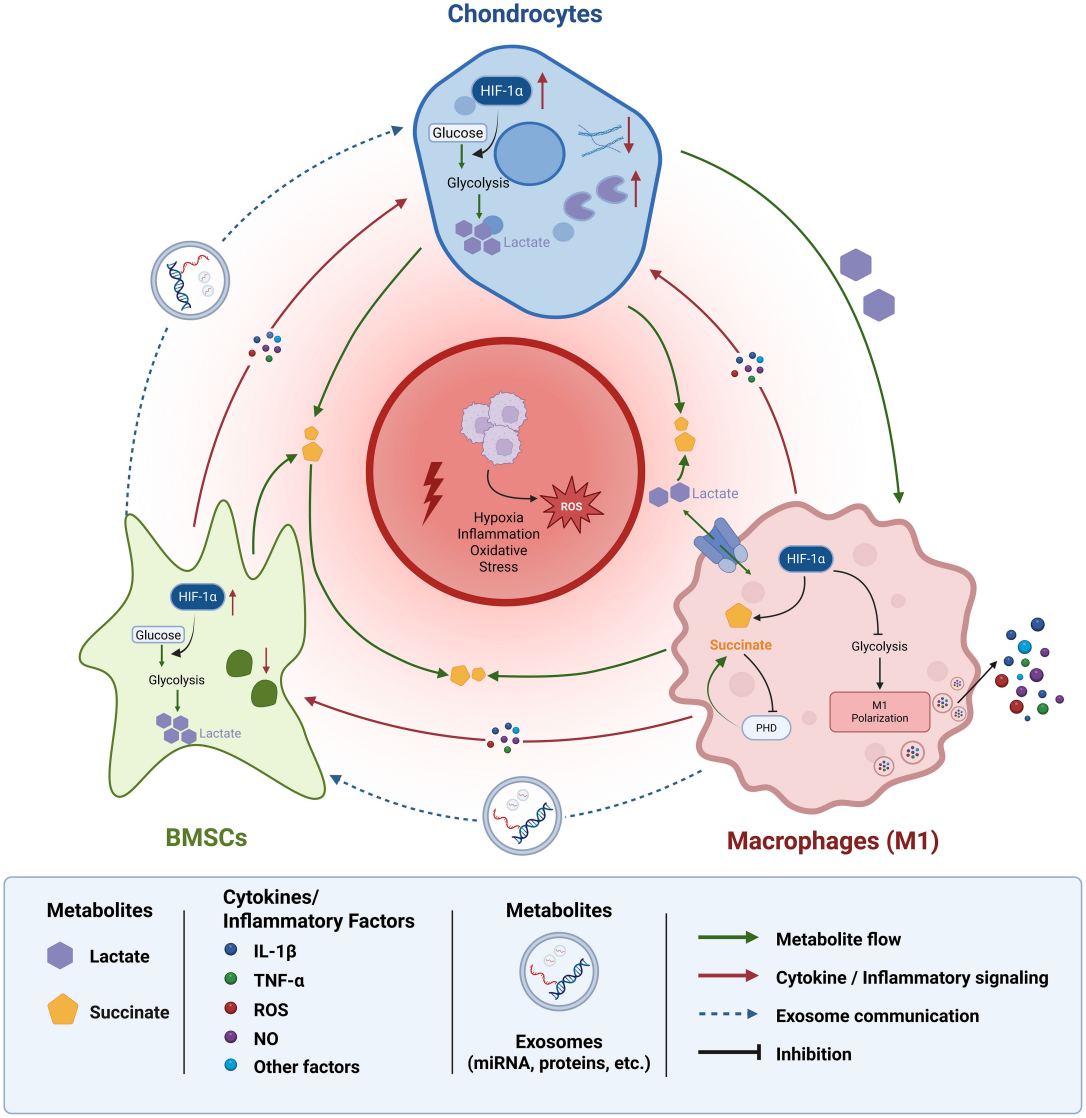
HIF-1α–centered metabolic crosstalk network orchestrates pathogenic interactions among chondrocytes, bone marrow mesenchymal stromal cells (BMSCs), and M1-polarized macrophages within the osteoarthritic joint microenvironment. Under combined stimuli—including hypoxia, pro-inflammatory cytokines, and oxidative stress—chondrocytes and BMSCs undergo HIF-1α–driven glycolytic reprogramming, characterized by increased glucose uptake, elevated glycolytic flux, and substantial lactate accumulation. Extracellular lactate serves dual roles: it can be imported by macrophages to fuel glycolysis and modulate intracellular signaling, and it can stabilize HIF-1α in an autocrine/paracrine manner—thereby reinforcing M1-like polarization in a glycolysis-dependent fashion. In turn, M1-polarized macrophages secrete a cascade of inflammatory mediators and immunometabolites, including IL-1β, TNF-α, reactive oxygen species (ROS), nitric oxide (NO), and succinate. These factors reciprocally impair chondrocyte viability and matrix synthesis, suppress BMSC chondrogenic differentiation, induce mitochondrial dysfunction, accelerate extracellular matrix (ECM) degradation, and amplify catabolic enzyme expression. Notably, succinate inhibits Prolyl Hydroxylase Domain protein (PHD) enzymes, thereby preventing HIF-1α degradation and potentiating a self-sustaining inflammation–metabolism feedback loop. Furthermore, extracellular vesicles—particularly exosomes—secreted by chondrocytes, BMSCs, and macrophages convey regulatory cargo such as microRNAs (miRNAs), metabolic enzymes, and signaling proteins. These vesicles may transfer regulatory cargo among joint-resident cells and thereby modulate local inflammatory and metabolic responses. However, the direction and magnitude of these effects depend on the cellular source and cargo composition of the vesicles, and several proposed intercellular links remain to be validated in human OA.

From a therapeutic perspective, engineered or metabolically preconditioned extracellular vesicles may provide a means of delivering protective miRNAs, proteins, or pharmacological agents to selected joint-resident cells (207–212). Nevertheless, extracellular vesicle-based therapy remains limited by heterogeneity in donor-cell sources and cargo composition, variation in isolation and characterization methods, uncertain biodistribution, production and standardization challenges, and insufficient long-term safety data (206–208, 213). Further studies are required to determine which extracellular vesicle populations are protective or detrimental in specific OA compartments and disease stages (**Figure 3**).

## 7 Therapeutic significance

### 7.1 Precision modulation of HIF-1α signaling

HIF-1α is considered a potential therapeutic target because it promotes the formation of hypoxic-injury responses and other adverse changes in OA (38). However, given that it has dual, context-dependent, and cell type-specific functions, direct targeting is not practical (155). Non-selective global inhibition of HIF-1α may suppress macrophage-driven inflammation but could also inhibit chondrocyte adaptation and BMSC-mediated repair (38, 115). Meanwhile, persistent HIF-1α activation may increase VEGF-A production and promote pathological angiogenesis, synovial inflammation, and pain-related neurovascular remodeling. This further supports the need for balanced regulation of HIF-1α, aiming to preserve its adaptive function in chondrocytes while limiting sustained HIF-1α/VEGF-A activation in pathological joint compartments (28, 29, 40). This is consistent with cartilage biology studies showing that HIF-1α is necessary for chondrocyte survival, glycolytic ATP production, and extracellular matrix synthesis under hypoxic conditions (25, 26). Therefore, complete inhibition of HIF-1α may impair the physiological adaptation of chondrocytes to the avascular cartilage microenvironment. Although the small-molecule HIF-1α inhibitor PX-478 has been shown to be anti-inflammatory and anti-arthritic in preclinical studies, systemically inhibiting it may interfere with the body’s adaptive and repair responses (214).

Therefore, a more appropriate strategy is to achieve precise regulation of HIF-1α signaling and reduce the excess of HIF-1α in macrophages to suppress glycolytic M1-type polarization and the production of inflammatory cytokines (215). Chondrocytes maintain a moderate level of HIF-1α activity to promote survival and mitochondrial quality control without excessive activation of glycolysis or catabolism (40, 144). The PHD/HIF regulatory axis may also require stage- and zone-specific consideration in articular cartilage. Although PHD inhibition can stabilize HIF-1α and potentially enhance hypoxic adaptation, Phd2 deletion in articular chondrocytes accelerates hypertrophic differentiation and reduces superficial zone thickness (87) Modulation of HIF-1α can promote the proliferation and chondrogenic differentiation of BMSCs, but excessive activation may induce senescence and an increase in lactate secretion (216, 217). Therefore, cell-specific delivery systems such as nanoparticles, hydrogels, antibody-conjugated carriers, and exosome-based platforms can be used to achieve spatial and temporal control (218, 219).

This principle is also relevant to the distinction between HIF-1α and HIF-2α. Previous studies suggest that HIF-2α can have a regulatory role in cartilage maturation through Runx2 destabilization, whereas OA studies indicate that sustained HIF-2α activation in adult articular cartilage is closely related to hypertrophic and catabolic responses (105, 106). Therefore, therapeutic strategies should not indiscriminately suppress hypoxia-inducible signaling, but selectively limit pathological HIF-2α activity while preserving appropriate HIF-1α-mediated adaptation.

### 7.2 Targeting downstream metabolic nodes

Considering the risk of non-specific inhibition of HIF-1α, targeting the downstream metabolic nodes may offer a more versatile therapeutic option (172, 220). Glycolytic enzymes and transporters, such as GLUT1, HK2, PFKFB3, PKM2, and LDHA, are all under the regulation of HIF-1α for metabolic reprogramming (221). Selective normalization of excessive glycolysis can reduce the accumulation of lactate and extracellular acidification; subsequently, matrix degradation and macrophage inflammatory activation are suppressed, and the necessary glycolytic activity for the survival of chondrocytes and BMSCs in a hypoxic environment is preserved (109).

Mitochondrial quality control and metabolite-mediated feedback loops are also important therapeutic targets (222). Selective HIF-2α inhibition may provide a complementary strategy. PT2385, a small-molecule HIF-2α inhibitor, has been shown to suppress HIF-2α and other downstream catabolic factors in inflammatory chondrocytes. A pH-responsive BSA-m-CQDs-PT2385 delivery system further improved cartilage penetration and reduced HIF-2α, ADAMTS4, and MMP13 expression throughout the cartilage layer *in vivo* (115). These findings support the therapeutic potential of targeting pathological HIF-2α activity, especially when such inhibition can be spatially restricted to degenerative cartilage. VEGF/VEGFR signaling may also be considered a downstream pathological effector of sustained HIF-1α- and HIF-2α-associated signaling. Local inhibition of VEGFR1/VEGFR2 signaling has been shown to reduce OA-related pain and cartilage degeneration, suggesting that targeting VEGF-mediated neurovascular and catabolic signaling may have therapeutic value in selected OA contexts (28). For example, promotion of mitophagy, restriction of ROS production, or regulation of the succinate/lactate signaling pathway suppress HIF-1α stabilization and inflammatory amplification (40, 223, 224). Rather than completely eliminating HIF-1α activity, these approaches aim to restore metabolic homeostasis and reduce pathological intercellular metabolic crosstalk in OA (24, 224).

### 7.3 Combination therapy and personalized metabolic intervention

Because OA progression is driven by interconnected inflammatory and metabolic pathways, combination therapy may be more effective than single-target intervention (225). This concept is supported by recent work on coordinated HIF modulation. A biomimetic gene vector was designed to co-deliver HIF-2α-targeting siRNA (siHIF-2α) and Mg2+ to inflamed OA joints. This strategy reduced HIF-2α expression, enhanced HIF-1α/SOX9-related cartilage repair signaling through Mg2+, decreased MMP-13 expression, and promoted macrophage repolarization toward an anti-inflammatory M2 phenotype (38). These findings further suggest that the balance between HIF-1α and HIF-2α may be therapeutically more important than targeting either pathway alone. Recent evidence from skeletal biology also suggests that activation of HIF-1 signaling may compensate for impaired oxidative phosphorylation in selected skeletal cell populations. Khan et al. reported that HIF-1 activation in periosteal cells mitigated the adverse effects of mitochondrial transcription factor A deletion and protected cortical bone formation under impaired oxidative phosphorylation (226). Although this study was not focused on articular cartilage, it supports the broader concept that HIF-1-mediated glycolytic adaptation may be beneficial in specific skeletal microenvironments and should be modulated in a cell type-specific manner. Anti-inflammatory agents may reduce cytokine-induced HIF-1α activation, whereas metabolic modulators may alleviate pathological glycolysis, mitochondrial dysfunction, and metabolite accumulation (43, 51). Together, these approaches may help disrupt the self-reinforcing inflammation–metabolism cycle that promotes OA progression (43).

BMSC-based therapy may benefit from metabolic preconditioning (227). Moderate hypoxic exposure or controlled HIF-1α activation can enhance BMSC viability, migratory capacity, and chondrogenic potential before transplantation (228, 229). However, excessive or prolonged HIF-1α activation may promote senescence and metabolic dysfunction, highlighting the importance of precise regulation (157, 230, 231).

Finally, omics-guided personalized medicine may help identify dominant pathological mechanisms in different OA subtypes (232). Single-cell sequencing, metabolomics, and spatial transcriptomics could support patient stratification and guide cell type-specific metabolic intervention, thereby improving the precision of disease-modifying OA therapy (233, 234). The potential therapeutic strategies targeting HIF-1α-related metabolic regulation in OA are summarized in **Table 2**.

**Table 2:** Potential therapeutic strategies targeting HIF-1α-related metabolic regulation in osteoarthritis.

Therapeutic strategy	Major target or intervention object	Mechanism of action	Potential therapeutic significance and consideration
Precise modulation of HIF-1α signaling	HIF-1α in macrophages, chondrocytes, and BMSCs	Regulation of HIF-1α activity based on cell type and pathological context, avoiding non-selective systemic inhibition	May suppress inflammatory polarization of macrophages, maintain hypoxic adaptation in chondrocytes, and promote BMSC-mediated repair; however, excessive inhibition or activation may produce adverse effects
Targeting downstream glycolytic nodes	GLUT1, HK2, PFKFB3, PKM2, and LDHA	Modulation of HIF-1α-regulated glycolytic enzymes and transporters to correct pathological glycolytic enhancement	May reduce lactate accumulation, extracellular acidification, matrix degradation, and inflammatory activation; basal glycolytic activity required for cell survival under hypoxia should be preserved
Regulation of mitochondrial quality control and metabolite feedback	Mitophagy, ROS, and succinate/lactate signaling pathways	Promotion of mitochondrial homeostasis, restriction of ROS production, and regulation of metabolite-mediated HIF-1α stabilization	May suppress inflammatory amplification and abnormal metabolic crosstalk, thereby restoring metabolic homeostasis in the OA joint microenvironment
Combined anti-inflammatory and metabolic intervention	Inflammatory pathways, abnormal glycolysis, mitochondrial dysfunction, and metabolite accumulation	Anti-inflammatory agents reduce cytokine-induced HIF-1α activation, whereas metabolic modulators correct pathological metabolic changes	May disrupt the self-reinforcing inflammation–metabolism cycle in OA progression; optimal combinations, dosages, and treatment windows require further investigation
Metabolic preconditioning of BMSCs	Hypoxic adaptation and HIF-1α activity in BMSCs before transplantation	Uses of moderate hypoxic exposure or controlled HIF-1α activation to enhance BMSC biological functions	May improve BMSC survival, migration, and chondrogenic potential; excessive or prolonged activation may induce senescence and metabolic dysfunction
Omics-guided personalized metabolic intervention	OA subtypes identified by single-cell sequencing, metabolomics, and spatial transcriptomics	Identification of dominant pathological mechanisms in different patients and guidance of cell type-specific metabolic intervention	May support patient stratification and precision therapy; reliable subtype criteria, predictive biomarkers, and clinical validation are still needed

HIF-1α, hypoxia-inducible factor-1α; OA, osteoarthritis; BMSCs, bone marrow mesenchymal stromal cells; ROS, reactive oxygen species.

## 8 Conclusion

HIF-1α is one of the key factors that coordinate the responses of hypoxia, inflammation, and metabolic stress in OA. The effects of HIF-1α vary in different cell types and locations; improper or excessive stimulation of HIF-1α may result in cartilage damage by increasing catabolism of chondrocytes as well as BMSC dysfunction and inflammation. Chondrocytes, BMSCs, and macrophages orchestrate to build a HIF-1α-centered metabolic network that promotes OA progression by amplifying metabolite-, cytokine-, ROS-associated, and exosome-mediated signals. This network may also involve HIF-1α-dependent VEGF-A elevation, which links hypoxic signaling to abnormal angiogenesis, vascular invasion, synovial inflammation, pain-related neurovascular remodeling, and disruption of the normally avascular cartilage niche. However, this network does not support the application of non-specific HIF-1α inhibition in therapeutic strategies. The role of HIF-2α in joint biology further supports this view, i.e., HIF-2α can inhibit Runx2-dependent chondrocyte maturation highlighting the context-dependent nature of HIF-2α signaling, whereas OA studies indicate that sustained HIF-2α activation in adult articular cartilage may promote hypertrophy, matrix degradation, VEGF-associated vascular remodeling, and osteochondral crosstalk. Taken together, these cartilage and skeletal biology studies indicate that HIF-1α is not simply a pathological mediator in OA. Instead, HIF-1α functions as a context-dependent regulator of hypoxic survival, glycolytic adaptation, mitochondrial oxygen consumption, extracellular matrix synthesis, collagen modification, and chondrocyte differentiation. The therapeutic goal should therefore be to restore balanced HIF-1α activity rather than to completely suppress or indiscriminately activate this pathway. In the future, strategies should focus on cell type- and stage-specific modulation of HIF-1α signaling, restoration of mitochondrial quality control, targeting of downstream metabolic nodes, and disruption of inflammatory-metabolic feedback loops.
